# Donor/Acceptor Photovoltaic Cells Fabricated on *p*-Doped Organic Single-Crystal Substrates

**DOI:** 10.3390/ma13092068

**Published:** 2020-04-30

**Authors:** Yusuke Yabara, Seiichiro Izawa, Masahiro Hiramoto

**Affiliations:** 1 Institute for Molecular Science, 5-1 Higashiyama, Myodaiji Town, Okazaki City, Aichi 444-8787, Japan; yabara@ims.ac.jp (Y.Y.); Izawa@ims.ac.jp (S.I.); 2 The Graduate University for Advanced Studies SOKENDAI, 5-1 Higashiyama, Myodaiji Town, Okazaki City, Aichi 444-8787, Japan

**Keywords:** organic single-crystal substrate, donor/acceptor junction, sheet conductivity, *p*-doped rubrene homoepitaxial layer

## Abstract

In this study, the operation of donor/acceptor photovoltaic cells fabricated on homoepitaxially grown *p*-doped rubrene single-crystal substrates is demonstrated. The photocurrent density is dominated by the sheet conductivity (σ_□_) of the *p*-type single-crystal layer doped to 100 ppm with an iron chloride (Fe_2_Cl_6_) acceptor. A 65 μm thick *p*-type rubrene single-crystal substrate is expected to be required for a photocurrent density of 20 mA·cm^−2^. An entire bulk doping technique for rubrene single crystals is indispensable for the fabrication of practical organic single-crystal solar cells.

## 1. Introduction

The creation of organic single crystals with high carrier mobility (reaching 40 cm^2^V^−1^s^−1^ [1,2,3,4]) was demonstrated in previous studies. Another recent investigation reported that a long exciton diffusion length (reaching 2–8 µm for rubrene single crystals) can produce a blend junction-free organic solar cell [5]. Several donor (D)/Acceptor (A) junctions using organic single crystals, such as the epitaxial growth of C_60_ (A) on single-crystal pentacene (D) [6], the organic solar cell between a C_60_ film (A) and a rubrene single crystal (D) (exhibiting an efficiency of 0.01% [7]), and the organic solar cell between fluorinated (A) and non-fluorinated copper phthalocyanines (D) single-crystalline nanoribbons exhibiting an efficiency of 0.007% [8], were also investigated. Building on these prior studies, we developed a doping technique for homoepitaxially grown rubrene single-crystal layers [9,10]. We aimed to create doped organic single-crystal wafers similar to doped silicon wafers. Initially, we fabricated an organic *pn*-homojunction photovoltaic cell on a doped rubrene single-crystal substrate [11]. We confirmed that the entire photocurrent generated under the *pn*-homojunction had a macroscopic area of 2 mm × 1 mm was collected through the *p*-doped homoepitaxial layer. However, the obtained photocurrent density under the intensified irradiation of the simulated solar light of 10 suns was only several μA·cm^−2^. We then fabricated organic donor/acceptor (D/A) heterojunction photovoltaic cells on doped rubrene single-crystal substrates. This was expected to generate a photocurrent of a larger magnitude due to the efficient exciton dissociation at the D/A heterojunction. As organic single crystals display high carrier mobility parallel to the crystal surface along the π-π stacking of organic molecules, a lateral-type organic D/A heterojunction cell was fabricated [11,12,13]. The main purpose of this study is to determine the variables required for the production of organic single-crystal substrates.

In this study, we demonstrate the operation of organic D/A heterojunction photovoltaic cells fabricated on *p*-doped rubrene single-crystal substrates. The sheet conductivity (σ_□_) of the *p*-type single-crystal layer dominated the photocurrent density.

## 2. Materials and Methods

**Figure 1:**
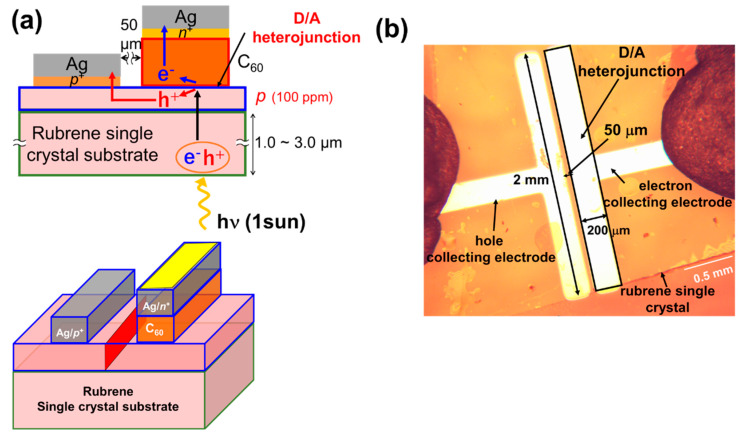
(**a**) Structure of rubrene single-crystal photovoltaic cell with a D/A heterojunction. A three-dimensional cell structure is also shown. The D/A heterojunction area is shown in yellow. The cross-sectional area of the p-layer is shown in red; (**b**) an optical microscopic image of a cell surface.

The structures of the donor/acceptor (D/A) photovoltaic cells fabricated on the *p*-doped organic single-crystal substrates are shown in Figure 1a with a diagram of the cell (Figure 1b). A *p*-doped single crystalline layer, which is homoepitaxially grown on a single crystal, acts as a *p*-type hole-transporting layer (Figure 1a, blue rectangular part). A D/A heterojunction is formed between the *p*-doped single crystalline layer acting as the donor, while a C_60_ film (Figure 1a, red rectangular part) acts as an acceptor. The excitons photogenerated in the crystal substrate diffuse to the D/A heterojunction and dissociate into electrons and holes. The electrons generated at the D/A heterojunction are collected vertically by an electron-collecting electrode (*n^+^*-C_60_/Ag). On the contrary, the holes generated at the D/A heterojunction are collected laterally through the *p*-layer (Figure 1a, blue rectangular part) to a hole-collecting electrode (*p^+^*-rubrene/Ag). Two types of areas can be defined in the present cell. One is the D/A heterojunction area (0.2 mm × 2 mm = 0.04 cm^2^) (Figure 1a, lower, yellow part), and the other is the cross-sectional area of the *p*-layer (20 nm × 2 mm = 4 × 10^−5^ cm^2^ for a *p*-layer thickness of 20 nm) (Figure 1a, lower, red part).

Single crystals of rubrene (Tokyo Chemical Industry) were grown by physical vapor transport in N_2_ (0.1 atm) [14,15,16]. The thicknesses of the crystals were approximately 2 μm. Using a vacuum evaporator housed in a glove box (EpiTech Inc., Kyoto, Japan), homoepitaxial films of rubrene were grown on a rubrene single-crystal substrate via depositing at a very low evaporation rate of 3.3 × 10^−3^ nm s^−1^ at room temperature [9,10]. The hole concentration (N) of the homoepitaxial film, measured by the Hall effect, is shown in Figure S1. For the undoped rubrene homoepitaxial layer, N is approximately 10^15^ cm^−3^, which corresponds to a purity of 10^−6^. Thus, the impurity concentration is less than 1 ppm. A homoepitaxial layer was doped with 100 ppm Fe_2_Cl_6_ by volume, which corresponds to a hole concentration (N) of 3 × 10^17^ cm^−3^. Doping was performed through co-evaporation with Fe_2_Cl_6_ (Sigma-Aldrich, 99.99%, Tokyo, Japan) [17,18] and Cs_2_CO_3_ (Sigma-Aldrich, 99.999%, Tokyo, Japan) [19,20] molecules used as the acceptor and donor dopants, respectively. The doping mechanism is summarized in [10] and in Figure S2. When using (Fe_2_Cl_6_) as an acceptor dopant, through electron transfer from the rubrene molecule to the acceptor molecule, a charge transfer (CT) state is produced (Figure S2c, right). The positive electric charge on the rubrene molecule is released thermally at room temperature. Consequently, the rubrene shows *p*-type behavior. The opposite mechanism for donor (Cs_2_CO_3_) doping is also shown in Figure S2c. A doping concentration of 100 ppm by volume corresponded to a dopant evaporation rate of 3.3 × 10^−7^ nm s^−1^. This was the result of reducing the evaporation rate using rotating disks with slits with an aperture ratio of 1:100. In order to form ohmic contacts for holes (Figure 1a), the *p^+^*-rubrene film (thickness: 10 nm) was doped with Fe_2_Cl_6_ at a concentration of 10,000 ppm and inserted between the Ag electrodes and *p*-type crystals. The *n^+^*-C_60_ film (thickness: 10 nm) was doped with Cs_2_CO_3_ at a concentration of 10,000 ppm and inserted between the Ag electrodes and C_60_ film to form ohmic contacts for electrons (Figure 1a). The lateral gap between the two electrodes was maintained at 50 μm (Figure 1b). The width of the D/A heterojunction (that is, the width of Ag electrodes) was maintained at 2 mm.

The current–voltage (*J–V*) characteristics were measured under the irradiation of simulated solar light (AM1, 100 mW·cm^−2^) (Asahi Spectra, HAL-320, Tokyo, Japan) from the rubrene substrate side. The sheet conductivity of *p*-doped rubrene single crystals was measured using a hole-only device (Figure S3). Surface images of the *p*-doped rubrene homoepitaxial layers were observed by atomic force microscopy (AFM, Seiko Instruments, SPI3800, Chiba, Japan). During the cell fabrication and measurements, none of the samples were exposed to air.

## 3. Results and Discussion

**Figure 2:**
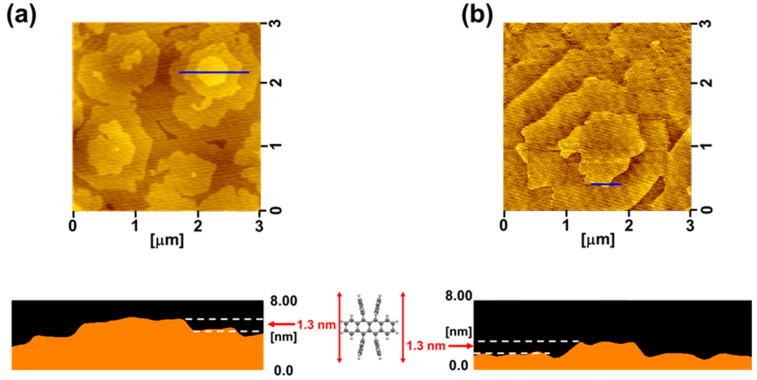
AFM images with cross-sectional profiles of 100 ppm Fe_2_Cl_6_ doped homoepitaxial layers grown on rubrene single-crystal substrates with a thickness of 20 nm (**a**) and 80 nm (**b**).

Figure 2 shows AFM images of the 100 ppm Fe_2_Cl_6_ doped rubrene films with thicknesses of 20 (Figure 2a) and 80 nm (Figure 2b). Both the 20 and 80 nm films showed many hexagonal structures oriented in the same direction. These tiny hexagonal structures have identical shapes to those of (001) rubrene single crystals with angles of 116° and 127° [21]. The observed step height of 1.3 nm from the cross-sectional profiles (Figure 2a,b) corresponds to a monomolecular step [21,22]. These observations clearly show that the 100 ppm Fe_2_Cl_6_ doped rubrene films grown on the rubrene single-crystal substrate are single crystalline homoepitaxial films, even for relatively thick films up to 80 nm. 

**Figure 3:**
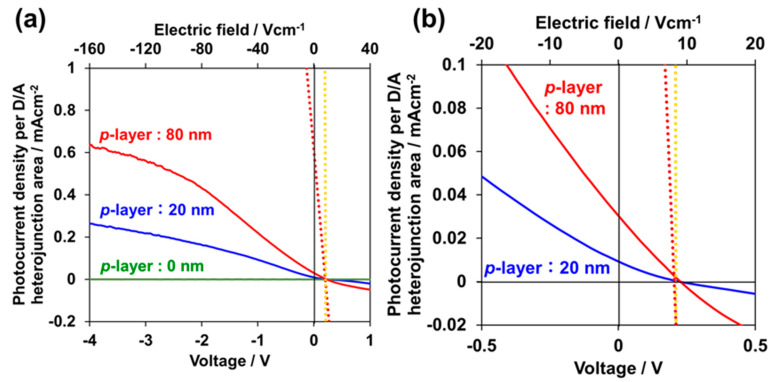
(**a**) *J–V* characteristics for cells with *p*-layer thicknesses of 0 (green), 20 (blue) and 80 (red). Simulated solar light of 100 mW·cm^−2^ was irradiated from the substrate side; (**b**) enlarged *J–V* characteristics of Figure 3a around the photovoltaic region (first quadrant). Attainable *J–V* curves for σ_□_ values of 7.9 × 10^−7^ (J_sc_: 0.6 mA·cm^−2^) and 3.1 × 10^−5^ S (J_sc_: 20 mA·cm^−2^) derived from the relationships in Figure 5 are shown by red and yellow dotted curves, respectively. On the upper horizontal axis, the electric field calculated for the distance between the right edge of the hole-collecting electrode and the right edge of the electron-collecting electrode are shown in Figure 1a.

Figure 3a shows the *J–V* characteristics in the voltage region between −4 and +1 V for rubrene single-crystal cells featuring D/A heterojunctions with *p*-layer thicknesses of 0 (green), 20 (blue), and 80 nm (red). At 0 nm (without the *p*-layer), little photocurrent was observed (Figure 3a, green curve). When the *p*-layer was deposited on the rubrene substrate, a stronger photocurrent was observed, and the photocurrent densities per D/A heterojunction area (see Figure 3a) reached significantly large values of 0.26 and 0.63 mA·cm^−2^ at −4 V for *p*-layer thicknesses of 20 and 80 nm, respectively (Figure 3a, blue and red curves). However, the observed short-circuit photocurrent densities (J_SC_) were significantly smaller than the saturated values of 0.0097 and 0.032 mA·cm^−2^ for *p*-layer thicknesses of 20 and 80 nm, respectively (Figure 3a,b, blue and red curves). The reported J_sc_ (0.04 mA·cm^−2^) and efficiency (0.01%) for a cell with a sandwich-type structure of Al/LiF/C_60_ (50 nm)/undoped rubrene single-crystal (several mm)/PEDOT:PSS (10 nm)/ITO [7] are comparable to 0.032 mA·cm^−2^ and 0.002% observed for the present lateral cell with an 80 nm-thick *p*-layer (Figure 3b). The external quantum efficiency (EQE) at −4 V reached 13% and 4% for the wavelength regions surrounding 350 nm and between 400 and 600 nm, respectively, for a *p*-layer thickness of 80 nm (Figure S4). These observations suggest that the magnitude of the potential photocurrent density of the D/A heterojunctions that appeared by applying reverse bias at −4 V represents the potential ability of the D/A heterojunction and is suppressed under a short-circuit condition. Figure 3b shows the enlarged *J–V* characteristics in the photovoltaic region. By increasing the *p*-layer thickness from 20 to 80 nm, J_SC_ increased by 3.2 times. This suggests that the observed suppression of the photocurrent density per D/A heterojunction area is due to the transport performance of the *p*-layer (Figure 1a, blue rectangular part). 

**Figure 4:**
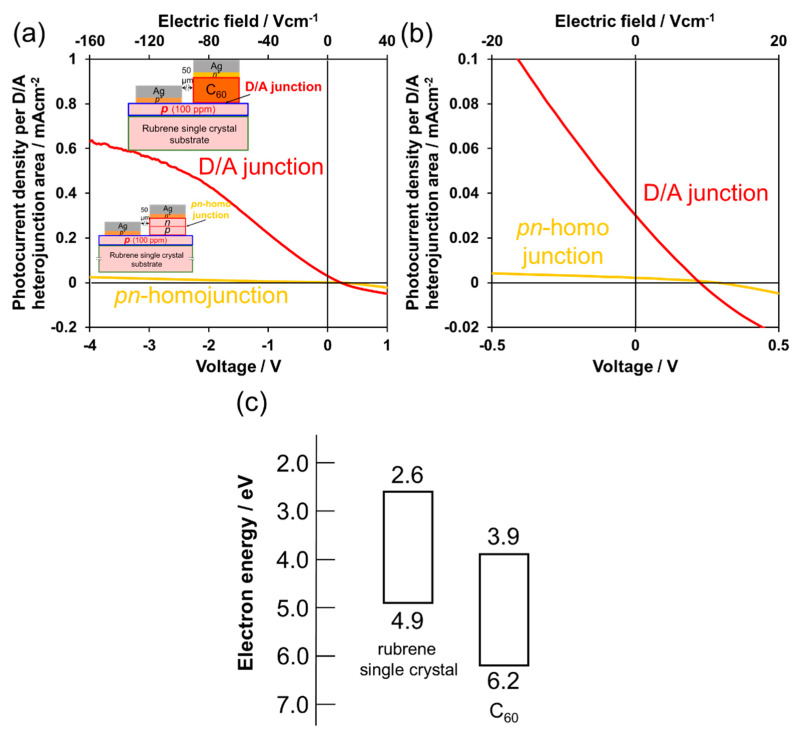
(**a**) *J–V* characteristics for cells with a *pn*-homojunction (yellow curves) and a D/A junction (red curves). The *p*-layer thickness of the rubrene single crystals are the same (80 nm). Simulated solar light of 100 mW·cm^−2^ was irradiated from the substrate side; (**b**) enlarged *J–V* characteristics of Figure 4a around the photovoltaic region (first quadrant). On the upper horizontal axis, the electric field calculated for the distance between the right edge of the hole-collecting electrode and the right edge of the electron-collecting electrode are shown (Figure 1a); (**c**) Energy diagram of C_60_ and rubrene single crystal.

Figure 4 shows a comparison of the *J–V* characteristics for our previously reported organic *pn*-homojunction cell [11] and the present D/A junction cell fabricated on the same *p*-doped rubrene single-crystal substrate. Through the introduction of the D/A junction, J_sc_ drastically increased by 15 times from 0.0021 to 0.032 mA·cm^−2^ due to photogenerated electrons and hole transfer at the energy offsets of the HOMO and LUMO formed at the C_60_/rubrene single crystal junction (Figure 4c). However, the 80 nm-thick *p*-doped rubrene single crystal layer appears to be unable to effectively flow laterally through the increased photocurrent. Therefore, the photocurrent still notably increased even under a short-circuit condition of 0 V and reached saturation at an approximate reverse voltage of −4 V (Figure 4a,b, red curve), while the photocurrent generated by the *pn*-homojunction remained low (Figure 4a,b, yellow curve). This observation suggests that the capacity for current flow through the 80 nm-thick *p*-doped rubrene layer is insufficient. However, since the electric field at a reverse bias of −4 V has a significantly small value of 1.6 × 10^2^ V·cm^−1^ due to the macroscopic distance of the lateral electrodes (0.25 mm, Figure 1b), we expect that this problem can be overcome by increasing the *p*-layer thickness of the rubrene single crystal.

**Figure 5:**
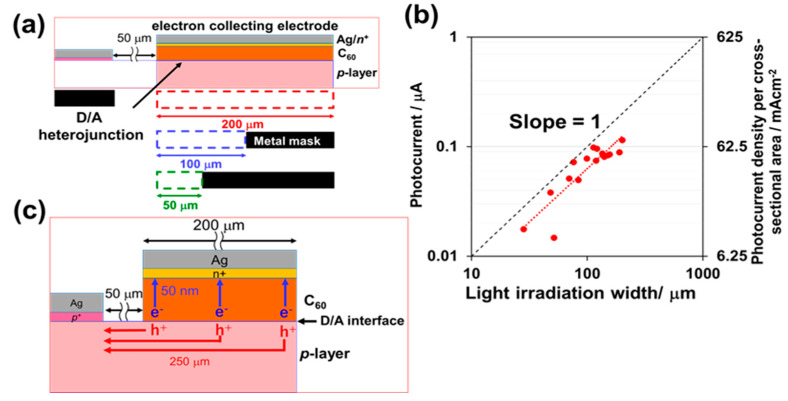
(**a**) Schematic of the light irradiation width experiments. The D/A heterojunction area was varied by inserting the metal mask into the light irradiation side; (**b**) double logarithmic plot between the observed photocurrent and light irradiation width (red dots). The short-circuit photocurrent density per cross-sectional area of the *p*-layer is shown on the right vertical axis; (**c**) schematic indicating hole transport through the *p*-layer.

First, we investigated whether the holes generated under the D/A heterojunction area (Figure 1a, blue rectangular part) are completely collected through the *p*-layer to the hole-collecting electrode. The light irradiation width on the D/A heterojunction area was varied by inserting a metal mask between the incident light and the crystal substrate (Figure 5a). Figure 5b shows a double logarithmic plot between the observed photocurrent and the light irradiation width where the slope is unified. The photocurrent is proportional to the light irradiation width. This result clearly shows that the photogenerated holes from the entire D/A heterojunction area were uniformly collected through the *p*-doped homoepitaxial layer irrespective of the macroscopic lateral distance from the right edge of the hole-collecting electrode (250 μm) (Figure 5c). Therefore, we concluded that the *p*-layer can be regarded as a pseudo-electrode. The photocurrent density per cross-sectional area of the *p*-layer reached a large value of 72 mA·cm^−2^ (Figure 5b, right vertical axis). Thus, we concluded that the *p*-doped homoepitaxial layer possesses considerable hole transport abilities.

**Figure 6:**
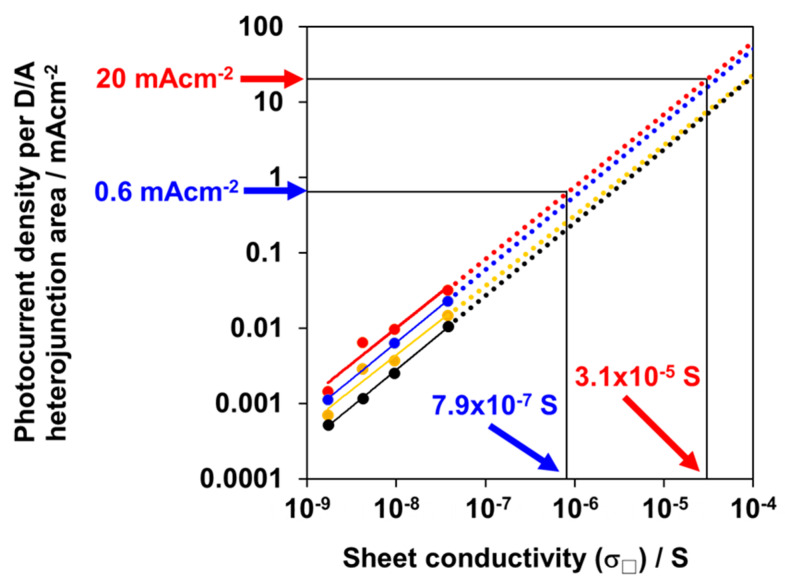
Double logarithmic plots between the photocurrent density per D/A heterojunction area and sheet conductivity of the *p*-layer (σ_□_) at the voltages of (**a**) 0 (red); (**b**) +0.05 (blue); (**c**) +0.10 (yellow); (**d**) +0.15 V (black). Dotted lines are the extrapolated lines. The σ_□_ values of 7.9 × 10^−7^ and 3.1 × 10^−5^ S, which correspond to the J_sc_ values of 0.6 and 20 mA·cm^−2^ at 0 V, are also shown.

Second, we attempted to confirm whether J_sc_ is dominated by the transport performance of the *p*-layer. Figure 5 shows the double logarithmic plots between the photocurrent density in the photovoltaic region (Figure 3b, first quadrant), including J_SC_ and the sheet conductivity of the *p*-layer (σ_□_), which was observed for the cells with various *p*-layer thicknesses. The slope of the J_SC_-σ_□_ relationship in the double logarithmic plot is unified—that is, J_SC_ is proportional to σ_□_ (Figure 6, red points). For other voltages in the first quadrant, the photocurrent densities are also proportional to σ_□_ at +0.05 (Figure 5, blue points), +0.10 (Figure 6, yellow points), and +0.15 V (Figure 6, black points). Evidently, the photocurrent density is dominated by the σ_□_ of the *p*-layer.

Conversely, a σ_□_ increase in the *p*-layer is expected to increase the photocurrent density in the photovoltaic region. As shown in Figure 6, J_SC_ = 0.03 mA·cm^−2^ is obtained at σ_□_ = 3.8 × 10^−8^ S. By extrapolation, to attain a J_SC_ of 0.6 mA·cm^−2^ (equivalent to the saturated photocurrent density for the 80 nm thick *p*-layer (Figure 3a), which represents the ability of photocurrent generation by the present D/A heterojunction (Figure 3a), a sheet conductivity σ_□_ of 7.9 × 10^−7^ S is needed. Moreover, according to the same extrapolation, to attain a J_SC_ of 20 mA·cm^−2^, which represents the typical photocurrent density of practical solar cells, a sheet conductivity σ_□_ of 3.1 × 10^−5^ S is needed. The attainable *J–V* curves, which are limited by the σ_□_ of the *p*-layers, for the σ_□_ values of 7.9 × 10^−7^ and 3.1 × 10^−5^ S derived from the relationships in Figure 6 are illustrated in Figure 3a,b by red and yellow dotted curves, respectively.

If we assume that the σ_□_-value is proportional to the *p*-layer’s thickness, the expected J_SC_ reaches 20 mA·cm^−2^ at a *p*-layer thickness of 65 μm. This thickness is close to the obtainable thicknesses of the rubrene crystals. Therefore, we conclude that all bulk-doped rubrene single crystals with a thickness of approximately 65 μm are required to construct practical organic single-crystal solar cells with a J_SC_ value of 20 mA·cm^−2^.

## 4. Conclusions

In conclusion, the operation of *p*-doped rubrene single-crystal photovoltaic cells with a D/A heterojunction was demonstrated. The J_SC_ values were revealed to be dominated by the sheet conductivity (σ_□_) of the *p*-type homoepitaxial layer. To attain a practical value of J_SC_ = 20 mA·cm^−2^, the *p*-layers should have a sheet conductivity (σ_□_) of 3.1 × 10^−5^ S. This is equal to the σ_□_-value of the entire bulk-doped rubrene single crystals (Fe_2_Cl_6_: 100 ppm) with a thickness of approximately 65 μm. In the future, we will focus on developing an entire bulk doping technique for organic single crystals for use in organic single-crystal photovoltaic cells.

## Supplementary Materials

The following are available online at https://www.mdpi.com/1996-1944/13/9/2068/s1, Figure S1: Hall effect, Figure S2: Energy diagram, molecular structure of dopant, and charge transfer state, Figure S3: Cell structure of hole only device, *J–V* curves, Figure S4: EQE and absorption spectra.
