# Supplementary material for: Donor/Acceptor Photovoltaic Cells Fabricated on p-Doped Organic Single-Crystal Substrates
Source: Materials (Basel). 2020 Apr 30;13(9):2068. doi: 10.3390/ma13092068 (PMC7254258; doi:10.3390/ma13092068)
Supplement: Supplementary file 1 [file materials-13-02068-s001.pdf]

Supplementary Information

Organic Single-Crystal Photovoltaic Cell Having Donor/Acceptor Junction

Yusuke Yabara[1], Seiichiro Izawa[1,2] and Masahiro Hiramoto[1,2*]

[1] Institute for Molecular Science, 5–1 Higashiyama, Myodaiji Town, Okazaki City, Aichi, 444–8787, Japan; yabara@ims.ac.jp(Y.Y); Izawa@ims.ac.jp (S.I.);

[2] The Graduate University for Advanced Studies SOKENDAI, 5-1 Higashiyama, Myodaiji Town, Okazaki City, Aichi, 444-8787, Japan;

*Correspondence: hiramoto@ims.ac.jp; Tel.: 0564-59-5536

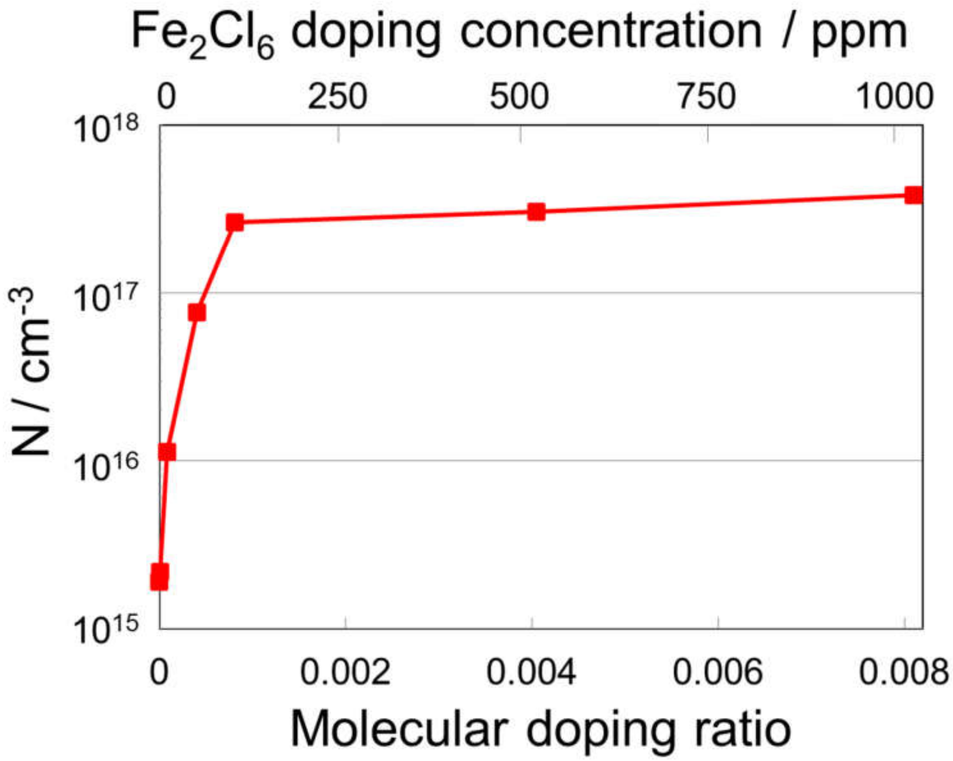

Figure S1. Dependence of hole concentration (N) measured by Hall effect on Fe_2Cl_6 doping concentration for rubrene single crystal [9].

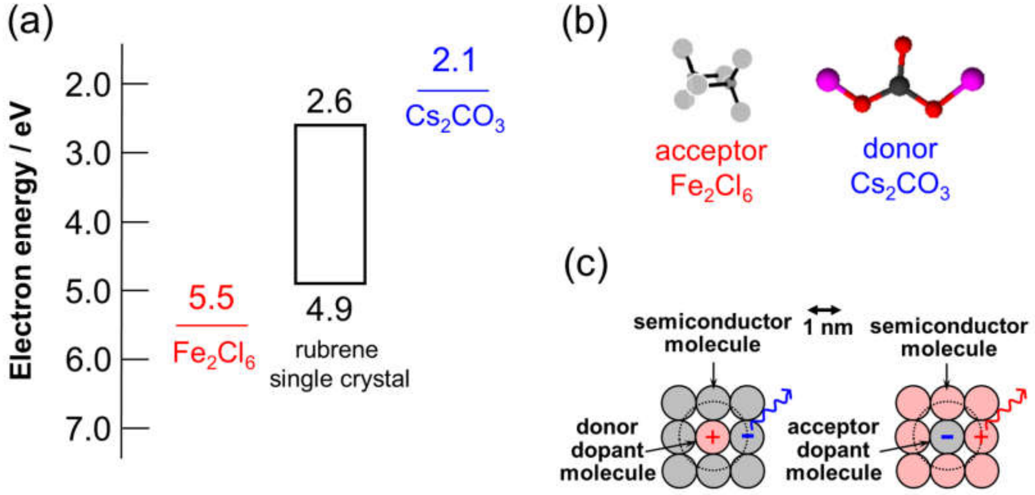

Figure S2. (**a**) Energy diagram of rubrene single crystal and Fe_2Cl_6 and Cs_2CO_3; (**b**) Molecular

structures of Fe_2Cl_6 and Cs_2CO_3. These molecules can vacuum-evaporated without decomposition; (**c**) Charge transfer (CT) states between donor molecules (Cs_2CO_3) (pink circles) and an acceptor molecule (Fe_2Cl_6) (gray circle). This CT state can be considered a CT exciton and a weakly bound electron around a positively ionized donor dopant molecule (left) and vice versa (right).

In the case of organic semiconductors, the behavior of dopants is not determined by their valence, as solid-state molecules are only bound by van der Waals forces, and no chemical bonds exist. Doping is caused by the charge transfer between the dopant molecule and the organic semiconductor molecule. Acceptor molecule (Fe_2Cl_6) (Figure S2b, left) and donor molecule (Cs_2CO_3) (Figure S2b, right) themselves are act as acceptor and donor. As shown in Figure S2a, the energy of the highest occupied molecular orbital (HOMO) of a donor dopant (Cs_2CO_3; 2.6 eV) should be less than that of the lowest unoccupied molecular orbital (LUMO) of the organic semiconductor molecule (rubrene; 2.6 eV). Upon electron transfer (ET) from the Cs_2CO_3 molecule (Figure S2b) to the rubrene molecule, a charge-transfer (CT) state is formed (Figure S2c, left). The negative electric charge on the rubrene molecule is thermally released at room temperature. Consequently, the rubrene shows *n*-type behavior. In contrast, as shown in Figure S2a, the energy of the LUMO of an acceptor dopant molecule (Fe_2Cl_6; 5.5 eV) should be higher than the energy of the HOMO of the host organic semiconductor molecule (rubrene; 4.9 eV). Upon ET from the rubrene molecule to the Fe_2Cl_6 molecule, a CT state is produced (Figure S2c, right). The positive electric charge on the rubrene molecule is released thermally at room temperature. Consequently, the rubrene shows *p*-type behavior.

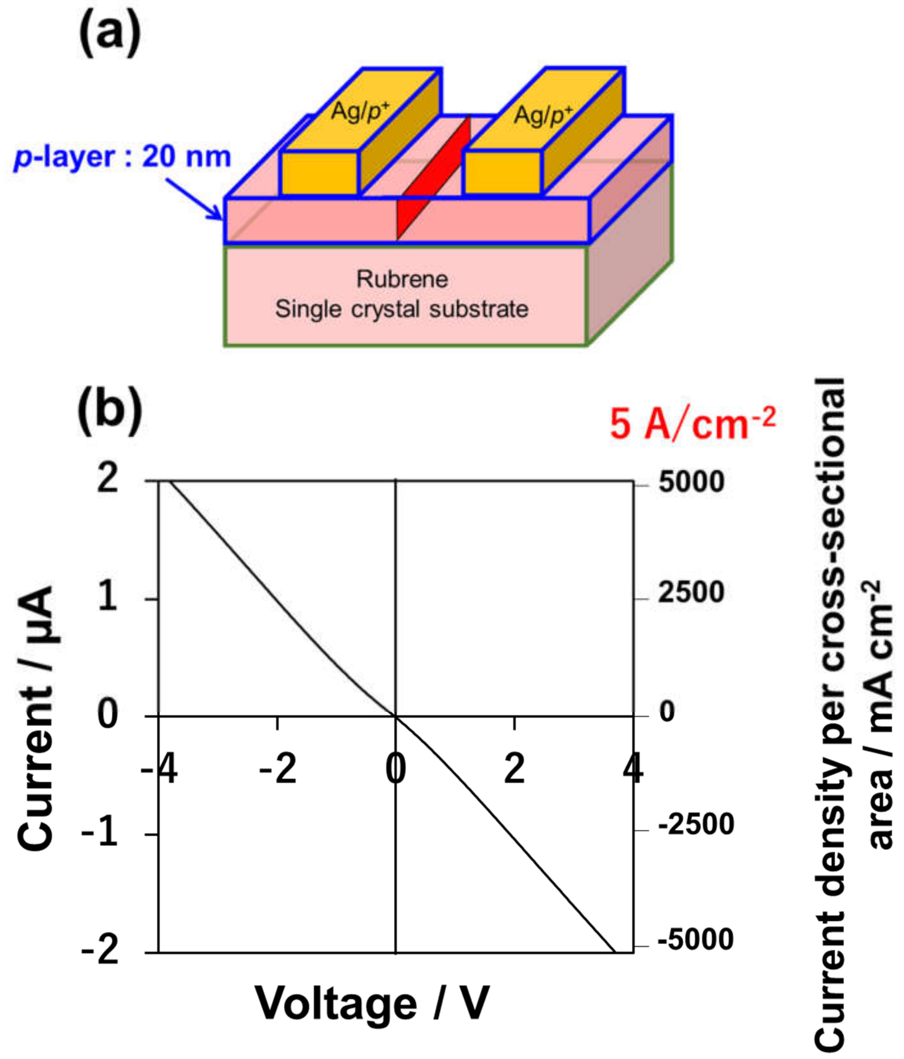

Figure 3. (**a**) Structure of hole-only device. The cross-sectional area of the p-layer is shown by a red rectangle; (**b**) Dark current–voltage (J–V) characteristics of a hole-only device with a p-type homoepitaxial layer of thickness 20 nm. Ohmic behavior was observed. The current density per cross-sectional area of the p-layer reached 5.3 Acm^{-2} at 4V (right vertical axis) and a sheet conductivity ($\sigma_{\square}$) of 9.5×10^{-9} S was obtained.

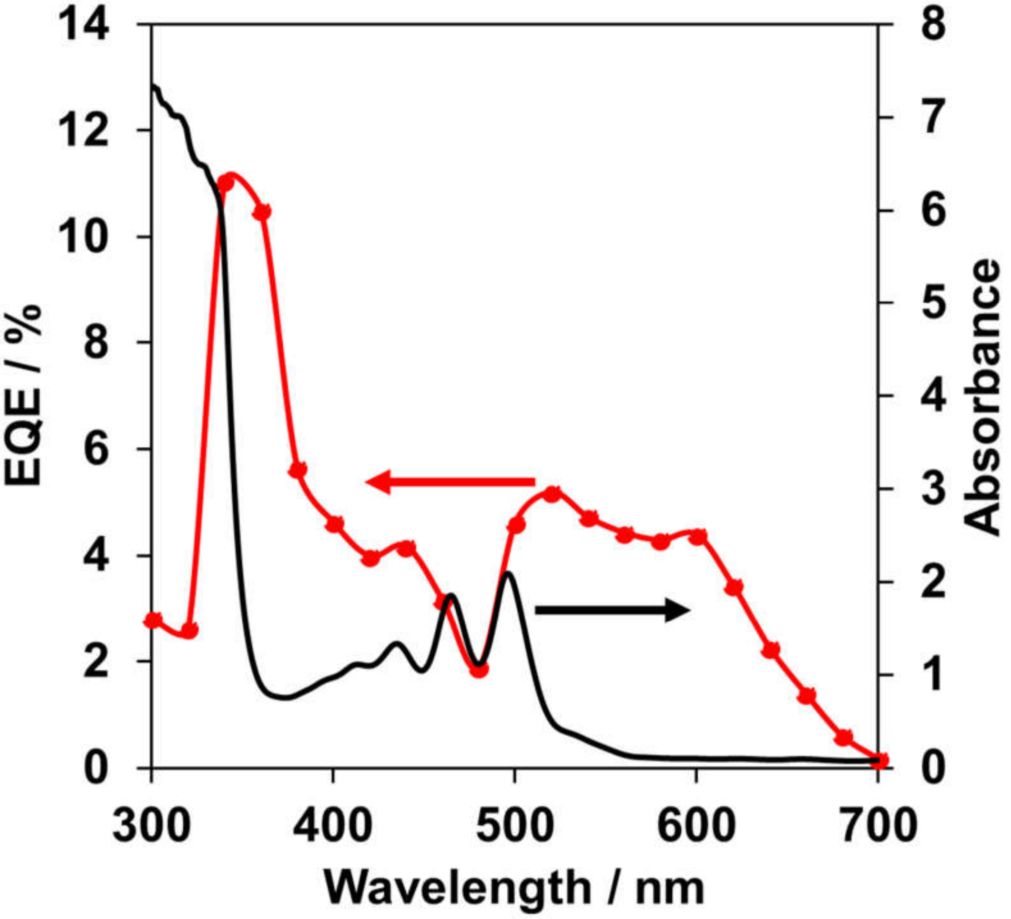

Figure 4. Action spectrum of external quantum efficiency (EQE) at −4V for the device with a p-layer thickness of 80 nm. Monochromatic light was irradiated from the crystal substrate side. EQE reached 13% and 4% for the wavelength regions around 350 nm and between 400 and 600 nm, respectively.
